# Spatio-temporal evaluation of social media as a tool for livestock disease surveillance

**DOI:** 10.1016/j.onehlt.2023.100657

**Published:** 2023-11-26

**Authors:** Samuel Munaf, Kevin Swingler, Franz Brülisauer, Anthony O'Hare, George Gunn, Aaron Reeves

**Affiliations:** a Division of Computing Science and Mathematics*,* University of Stirling*,* Stirling*,* United Kingdom; b Centre for Epidemiology and Planetary Health, Department of Veterinary and Animal Sciences, Northern Faculty, Scotland's Rural College (SRUC), Inverness*,* United Kingdom; c SRUC Veterinary Services, Scotland's Rural College (SRUC), Inverness*,* United Kingdom; d Centre for Applied public health research, RTI international, Raleigh*,* NC*,* USA

**Keywords:** Veterinary epidemiology, Disease surveillance, Infodemiology, Infoveillance, Time series, Social media, Spatio-temporal, Anomaly detection, Avian flu

## Abstract

Recent outbreaks of Avian Influenza across Europe have highlighted the potential for syndromic surveillance systems that consider other modes of data, namely social media. This study investigates the feasibility of using social media, primarily Twitter, to monitor illness outbreaks such as avian flu. Using temporal, geographical, and correlation analyses, we investigated the association between avian influenza tweets and officially verified cases in the United Kingdom in 2021 and 2022. Pearson correlation coefficient, bivariate Moran's I analysis and time series analysis, were among the methodologies used. The findings show a weak, statistically insignificant relationship between the number of tweets and confirmed cases in a temporal context, implying that relying simply on social media data for surveillance may be insufficient. The spatial analysis provided insights into the overlaps between confirmed cases and tweet locations, shedding light on regionally targeted interventions during outbreaks. Although social media can be useful for understanding public sentiment and concerns during outbreaks, it must be combined with traditional surveillance methods and official data sources for a more accurate and comprehensive approach. Improved data mining techniques and real-time analysis can improve outbreak detection and response even further. This study underscores the need of having a strong surveillance system in place to properly monitor and manage disease outbreaks and protect public health.

## Highlights

• Tweet volume spikes visually correspond to avian flu case surges.
• Weak temporal correlation observed between tweet count and official cases.
• Spatial analysis reveals disease awareness concentration in specific regions.
• Bivariate Moran's I analysis highlights weak, local correlation.
• Social media valuable for sentiment analysis, but insufficient for standalone surveillance.

## 1 Introduction

In the summer of 2020, an emerging outbreak of Avian influenza (AI) subtype H5N8 was detected in Russia and Kazakhstan [1]. Wild and migratory birds were determined to cause the outbreak, and thus subsequently spread into Northern Europe through the nomadic nature of waterbirds (i.e., ducks, swans, and geese). This further culminated in the virus being found in poultry farms in the Netherlands, Germany, Belgium, and France by October 2020, resulting in mass culling of livestock. As predicted by epidemiologists, the spread eventually reached the United Kingdom by Late November/Early December 2020 with Highly pathogenic AI being found in rearing turkeys in Northallerton, North Yorkshire and subsequent biosecurity measures being implemented by the Animal and Plant Health Agency (APHA) [2].

Contemporary surveillance systems struggle to factor in the influence of wild animal populations mixing with domesticated livestock populations, therefore creating vessels for disease transmission that is difficult to prevent without sound biosecurity measures [3]. This can be exacerbated by the type of farm (commercial, single-species, open air, mixed). These biosecurity measures can only be brought about if robust early warning signals are in place, to alert farmers and backyard keepers that a threat is imminent or highly likely.

Modeling this from a spatio-temporal perspective paints a clearer picture of spikes in activity and can assist in early disease detection [4]. Literature indicates that this can be effective in larger livestock animals, such as pigs and cattle, however, still remains a challenging prospect with poultry and birds. Social media signals related to AI are usually confirmed outbreaks through governmental agencies, which are cascaded through the networks and often retweeted among farming communities [5].

Developments in social media platforms have supplied the public with an immense selection of topical information and the ability to communicate globally [6]. Computational methods related to text analysis and data mining of such internet data have promptly increased in both applicability and usefulness across an array of domains. Such methods have been adopted extensively in the human health field, especially regarding using patient triage comments to predict the progression of illness/disease [7].

However, within the veterinary domain, its application remains rather scarce, as the data isn't as dynamic nor voluminous. Work conducted by Robertson and Lee in 2016 first highlighted the efficacy of social media data for AI risk surveillance in North America, whereby they devised anomaly detection models in a time series manner, to pinpoint possible instances of early disease detection. By adopting a spatio-temporal approach, they could highlight clusters of locations which displayed signs of unusually large twitter activity related to AI specific terms and phrases [8].

Regarding social media as a tool for surveillance systems, literature in the UK and even Europe is scarce, with only brief feasibility studies being conducted in North America by Robertson & Lee (2016). Google was one of the first social media tools to be utilised over a decade ago and has proven to be a popular tool for health researchers to use search term frequencies for influenza tracking [9]. Spikes in search history related to viruses and their subsequent symptoms/clinical signs can be monitored in a time series manner and drilled down for locations to obtain a granular picture of the disease spread. This method was then expanded to adopt other forms of social media platforms and web scraping techniques [10].

### 1.1 Study aim

This paper looks to address the gap in existing veterinary research by combining the fields of computer science with veterinary epidemiology to supplement traditional surveillance methods through the usage of social media data. The aim of this study is to explore the effectiveness of Twitter as a potential surveillance tool in detecting future outbreaks of AI in the UK, through matching tweets with spatio-temporal epidemiology of AI.

## 2 Methodology

### 2.1 Study design

This research retrospectively examined a cohort of UK Twitter users who had tweeted about AI during the years 2021 and 2022. The goal of this study was to determine whether a Twitter search could match the recognised Spatio-temporal patterns of the AI virus without reading or analysing the content of the tweets.

### 2.2 Data collection

#### 2.2.1 Twitter dataset

The Twitter search API was activated in Python through the Tweepy (www.tweepy.org) module, with developer-level access being obtained which permits the extraction of up to ten million tweets per month. A retrospective approach was applied on the data extraction, with the date range 1st January 2021 to 31st December 2022 being selected as the time filter.

Multiple variations of the term “avian influenza”, including spelling variations and colloquial denotations (i.e. bird flu) were used as the keyword search terms, in addition to subtypes (i.e. H5N1). To ensure a relatively even distribution of extracted tweets across the year and given the API restrictions, 24-time intervals were allocated, representing each month of the year, and 500 tweets (Twitter API maximum was between 10 and 500) were collected for each interval. Therefore 12,000 tweets were retrieved in the first instance, and after the UK location filter was applied, this was reduced to 5843 unique tweets (2566 in 2021 and 3277 in 2022).

#### 2.2.2 Confirmed cases dataset

Official AI confirmed cases for the years 2021 and 2022 were extracted from the APHA Twitter page.

[11]. This was performed using a string match whereby every instance of the phrase, “Highly pathogenic avian influenza H5N1 has been confirmed” was extracted.

After a thorough review, we identified 82 unique outbreak occurrences in 2021 and 218 in 2022, giving a total of 300. Upon eliminating 5 locations that could not be converted into geographical coordinates, we retained information on 295 distinct outbreak events.

### 2.3 Geolocation extraction

Free text location data was converted into geographic coordinates in our study using the Python GeoPy package (latitude and longitude). Using OpenStreetMap's geocoding service, this procedure, also known as geocoding, was carried out. We were able to perform spatial analysis and visualise the data on maps by converting the text-based location data into standardized geographic coordinates. This allowed for a more thorough understanding of the spatial distribution of tweets and their relationship to confirmed disease outbreak cases**.**

### 2.4 Data analysis

To determine the relationship between Twitter activity and confirmed cases, a spatio-temporal analysis was conducted.

#### 2.4.1 Temporal analysis

Firstly, a time series approach was utilised to portray the related AI activity over the span of the time period and highlights relevant periods of activity through spikes in frequency.

Tweets were aggregated on a daily basis to match the timeline from the confirmed cases, with the aim of visually emphasising the overlaps between the two datasets. The temporal structure was calculated through the standard time series modeling approach via summary statistics, Autoregressive Integrated Moving Average (ARIMA), Seasonal Autoregressive Integrated Moving Average (SARIMA) and the Autocorrelation function through the Box-Jenkins approach.

To determine the strength and statistical significance of the correlation between tweet counts and confirmed case counts, the Pearson correlation coefficient (r) was calculated.

#### 2.4.2 Spatial analysis

Exploratory spatial data analysis was utilised to investigate the regional relationship between the incidence of confirmed cases and tweets.

Moreover, to assess the spatial autocorrelation between the standardized tweet count and standardized case count of AI, bivariate Moran's I analysis was executed [12]. We computed a local version of Moran's I that is interpreted as a z-score, not restricted to the −1 to 1 range, unlike global Moran's I.

## 3 Results

By assessing both the geographical and chronological aspects of the data, our objective was to comprehend how public interest and awareness shifted across various regions and timeframes. This investigation provides essential insights into the public's reaction to the AI outbreak and underlines the potential of social media platforms as a monitoring tool for tracking disease-related conversations.

### 3.1 Temporal analysis

**Fig. 1:**
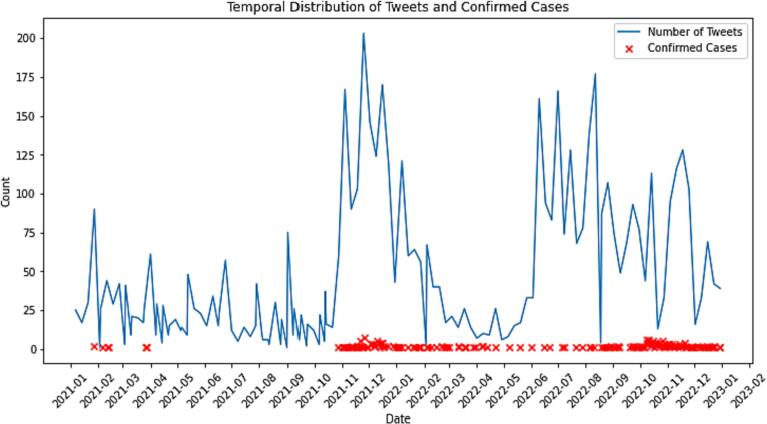
Temporal distribution of tweets and confirmed cases.

The results presented in Fig. 1 depict the temporal distribution of tweets and confirmed case. A visual relationship between the two variables is observed, indicating an increase in public attention and awareness during periods with higher confirmed cases.

In the first two months of 2021, January and February, there were notable spikes in tweeting activity corresponding to the emergence of some confirmed cases (highlighted by the red crosses). This pattern suggests that the public was actively discussing and sharing information about the AI situation in the UK during these months. Following a relatively quiet period from April 2021 to September 2021.

However, the most significant spikes in both tweeting activity and confirmed cases occurred in November and December 2021. This period witnessed the highest number of cases, which in turn led to increased public discourse on social media platforms. The sharp increase in tweets during these months suggests a heightened sense of urgency and concern among the public regarding the outbreak.

This continued in to the first quarter of 2022, with tweeting activity quelling after March, followed by a resurgence after May. Frequent oscillations occurred from May until the end of the year, with the largest sustained activity occurring from the second half of the year 2022.

Overall, the results demonstrate a strong association between the prevalence of cases and the level of public engagement on social media. This relationship highlights the importance of monitoring social media activity in understanding public sentiment and awareness during disease outbreaks.

**Fig. 2:**
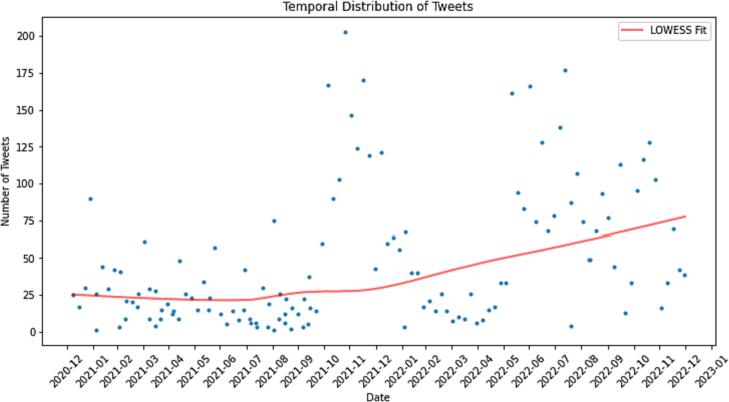
Temporal distribution of tweets with LOWESS fit.

Fig. 2 displays the number of tweets for each day, and a LOWESS fit line is drawn to show the underlying trend in the data. The LOWESS fit line, depicted in red, smooths out the daily fluctuations and highlights the general trend in the tweeting activity. The plot clearly highlights the significant increase in tweet frequency as time progressed.

The results from the SARIMAX (Seasonal AutoRegressive Integrated Moving Average with eXogenous regressors) model produced estimated coefficients for the AR and MA terms as −0.1039 and − 0.5385, respectively.

The AR coefficient was not statistically significant, as its *p*-value (0.47). This suggests that the autoregressive term does not have a strong influence on the time series. In contrast, the MA coefficient displayed a significant p-value indicating that the moving average term plays a more critical role in the time series.

### 3.2 Pearson's correlation coefficient

The Pearson correlation coefficient (r) statistic obtained was 0.104, indicating a weak positive correlation between the number of tweets and confirmed cases. Subsequently, this highlights that the relationship between tweeting activity and confirmed cases is not strong, and changes in one variable do not appear to have a substantial effect on the other.

The associated *p*-value was greater than the commonly used significance level of 0.05, meaning that the correlation wasn't statistically significant.

Based on these results, it can be inferred that there is no strong evidence to suggest a substantial relationship between the number of tweets and confirmed cases.

### 3.3 Spatial analysis

Exploratory analysis as shown in Table 1, indicates that the majority of the tweets discussing AI were posted from the users who stated “United Kingdom” as their main location, with 615 tweets originating from this location. London, the capital city, had the second-highest tweet frequency, accounting for 405 tweets. This is not surprising given London's large population and status as a global city, where public interest in health-related topics is expected to be higher.

**Table 1:** Top 10 locations by tweet and confirmed case frequency.

Tweet location	Count
United Kingdom	615
London	405
England	238
Scotland	219
Wales	91
Northern Ireland	54
North West, England	42
Edinburgh, Scotland	42
Somerset	40
South West, England	40
Confirmed case Location	Count
Attleborough	13
Thirsk	11
Dereham	9
Alford	9
Mundford	6
Wymondham	4
Much Hoole	4
Redgrave	4
Heybridge	4
Taverham	4

These findings suggest that public interest and awareness of the disease are more concentrated in specific regions within the UK. Urban areas such as London and Edinburgh seem to have a higher tweet frequency, which could be attributed to their larger populations and more extensive media coverage of the issue.

In addition, the locations for the confirmed cases were much more precise, given the official confirmation by APHA. These locations were considerably more rural and not in close vicinity with major cities.

**Fig. 3:**
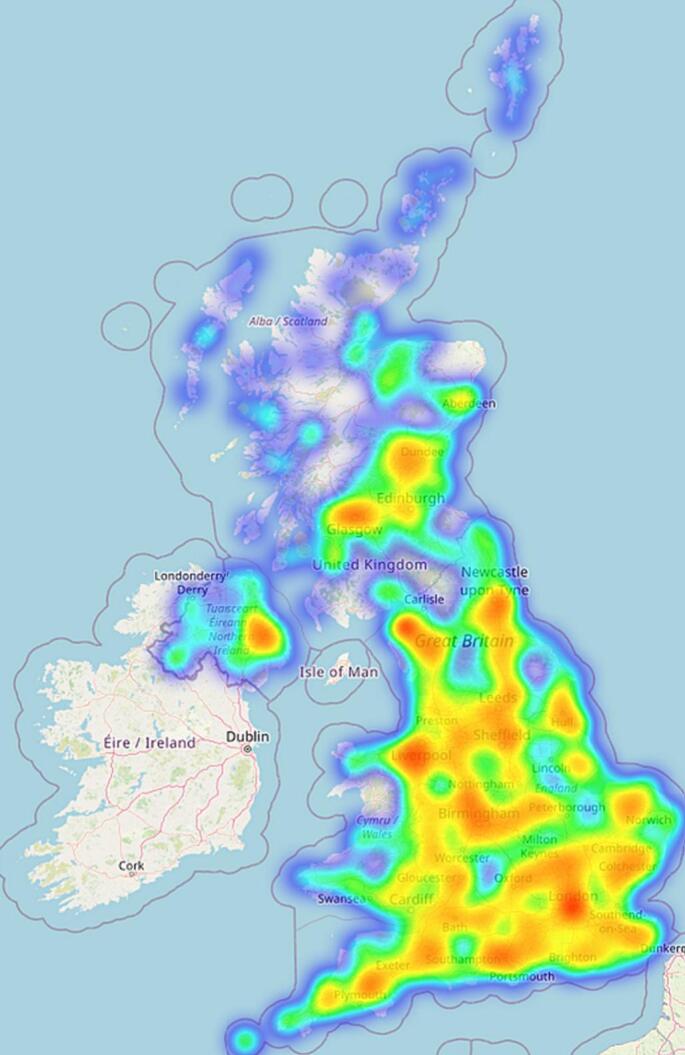
Tweet heatmap.

Fig. 3 depicts the density of the tweet locations, with London and Edinburgh being the two cities with the highest densities. Th map displays a large variance of locations spanning the entire UK, including some points within rural areas such as the Scottish Highlands and islands.

**Fig. 4:**
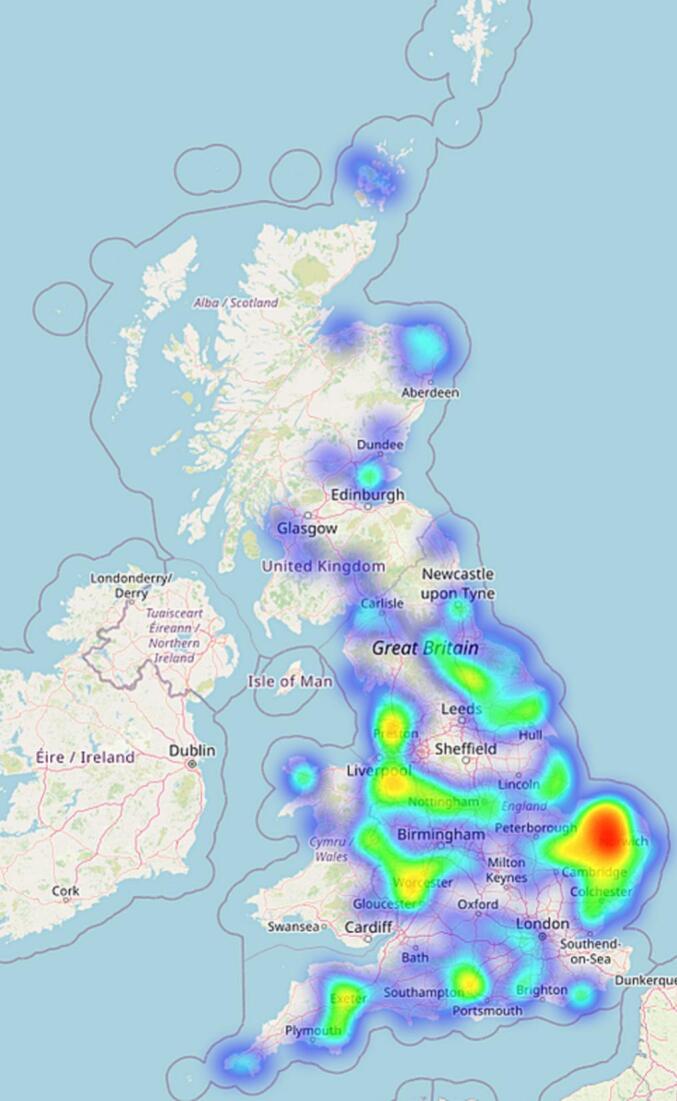
Confirmed cases heatmap.

In contrast, the confirmed cases as visualised in Fig. 4 are more concentrated within the rural areas of England, such as Norwich.

**Fig. 5:**
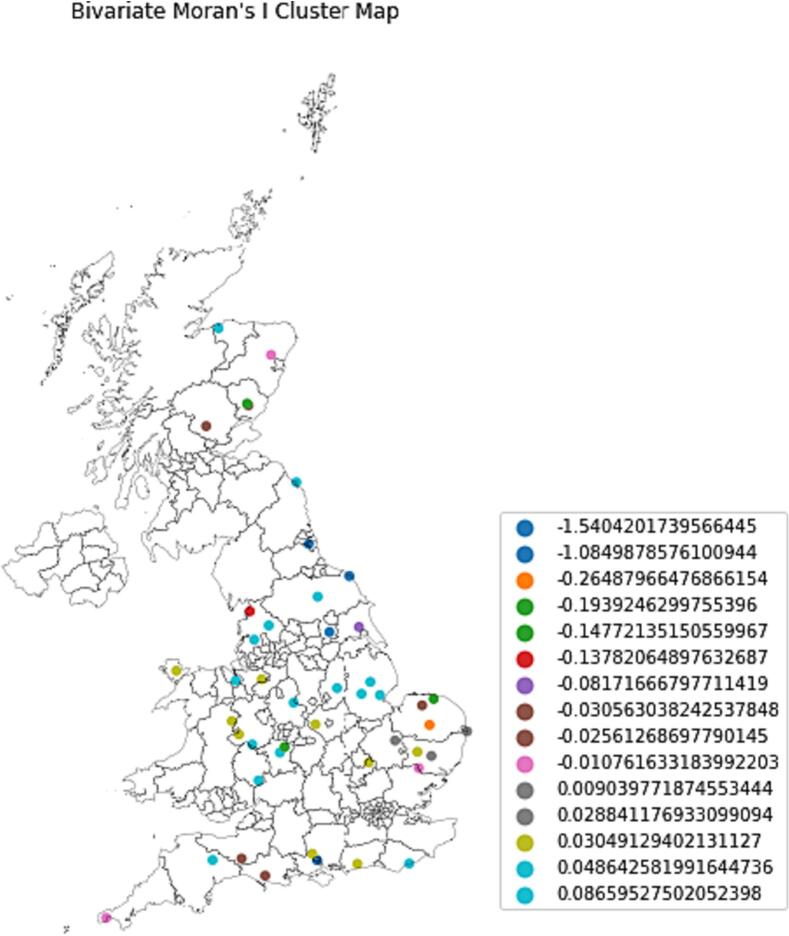
Bivariate Moran's I cluster map.

Fig. 5 shows a Bivariate Moran's I cluster map that visually represents the spatial autocorrelation between confirmed cases and tweets across the UK. The mapping of these values helps comprehend the spatial diversity in the correlation between tweet counts and case counts throughout the study region.

Spatial correlation between tweet counts and case counts varies between locations, as shown by the range of bivariate Moran's I values from −1.54 to 0.086. The z-score measures how many standard deviations a value is from the mean and determines its position relative to it.

At Hampshire and Tyne & Wear, both tweet count and case count deviate from their averages with a value of −1.54, indicating a significant local trend that opposes the general pattern.

On the contrary, the value 0.086 indicates a small positive spatial correlation, just above the average, between the count of tweets and cases in many areas across the UK that is most aligned with neighbouring values. The pattern appears to be slightly more congruent than what a random distribution would suggest.

The majority of values between 0.03 and 0.08 indicate a slight positive spatial autocorrelation between tweet counts and case counts at various locations. The z-scores being close to the mean suggest that the tweet and case counts at these locations are not significantly different from the overall average in the dataset. Thus, it can be deduced that these regions do not deviate significantly from the observed pattern in the data and are more or less in line with it. The discovery suggests that as the number of confirmed cases increases, so does the number of tweets about avian flu in those regions and vice versa. Though present, the weak positive correlation indicates that the relationship is not very strong or consistent across these areas. Other factors may also have an impact on the number of tweets and confirmed cases.

## 4 Discussion

This study looked at the correlation between tweets regarding Avian influenza and cases that were officially confirmed in the UK in 2021 and 2022. Furthermore, we aimed to test the viability of using social media, specifically Twitter, as a surveillance tool for disease outbreaks, through conducting temporal, spatial, and correlation analysis.

The temporal analysis displayed that there were periods of increased tweeting activity that coincided with some confirmed cases of avian flu. The correlation between the quantity of tweets and the number of confirmed cases was, however, weak and statistically insignificant, according to the Pearson correlation coefficient. This suggests that counting tweets alone may not be a reliable way to keep track of avian flu outbreaks. The geographic distribution of tweets and confirmed cases was revealed by the spatial analysis. Additionally, a weak negative spatial correlation that was not statistically significant was revealed by the bivariate Moran's I analysis. These results provide additional evidence that there are drawbacks to using social media data as a surveillance tool for avian flu outbreaks.

With regard to the efficacy of social media as a useful data source, Twitter appears to be the most adept software when conducting real-time analysis. The 280-character limit also acts as an incentive for concise and informative messages, partially ridding the content of unnecessary noise. Hashtags also expediate information flow as communities of like-minded individuals are created immediately through the sharing of a topic (e.g. #birdfluUK). The content of the tweets derived in this study would indicate the majority of the textual substance falls under public health communication and awareness. This is reinforced by the spikes in frequency of topics discussed after the onset of a confirmed case, as opposed to before.

This awareness could act as a catalyst for improved animal health planning. The main difference between “awareness-oriented” messages and “outbreak-oriented” messages are the more volatile fluctuations and short-term frequencies associated with the former [8]. The Ebola outbreak in West Africa in 2014 is a prime example of social media being used as a purveyor of public health awareness [13]. Extracting and analysing this data to better understand the content within these messages can play a vital role in assessing public perceptions and enhancing veterinary health guidelines.

### 4.1 Influence of COVID-19 pandemic on online discourse

Regarding the timing of the survey during the COVID-19 pandemic, we acknowledge that the global focus on infectious diseases may have impacted social media conversations. Studies indicate that social media played a crucial role in distributing COVID-19 information, with both positive and negative effects [14,15]. Numerous studies provide evidence of COVID-19's influence on social media discussions about animal diseases. For instance, Aria et al. (2022) highlighted that the pandemic caused a rise in social media usage for sharing experiences and seeking information, impacting discussions on diverse subjects, including animal diseases [16]. Jafarinejad et al. (2021) made a similar observation, stating that the pandemic in 2021 highlighted how social discourse underwent extreme changes, impacting community morale and polarisation, with potential implications for discussions [17].

It is essential to consider the study's methodological robustness, which includes controls to distinguish between human and animal health discussions, in this context. The global impact of the pandemic extended beyond human infectious diseases and may have impacted conversations about animal diseases on social media. The study aimed to reduce these overlaps by focusing on avian flu search terms, an important aspect of livestock disease surveillance and thus intended to eliminate general discussions about human health issues, including pandemic-related ones. By taking this approach, we ensured the data collected truly reflected concerns and trends in livestock disease, largely unaffected by the dominant COVID-19 narrative. However, it must be noted that extricating specific avian flu outbreak information from discourse regarding the pandemic is difficult in its entirety, particularly during the years 2021 and 2022, although this may potentially be curtailed by bringing in other modes of social media, namely livestockforums.

### 4.2 Limitations

It is essential to evaluate the limitations of this analysis. Firstly, the data only represents tweets and may not accurately reflect the overall public sentiment or concern about the Avian flu. Secondly, the spatial distribution of tweets might be influenced by factors such as population density and internet access, which could lead to an overrepresentation of tweets from urban areas. Lastly, the results are based on a specific time frame and may not account for changes in public interest or awareness over time.

Despite these limitations, the spatial analysis provides valuable information that can help public health authorities and policymakers target their communication strategies and interventions more effectively. By focusing on areas with a higher tweet frequency, they can better address public concerns and raise awareness about Avian flu prevention and control measures.

## 5 Conclusion

In conclusion, the limitations of using social media data, particularly Twitter, as a stand-alone surveillance tool for disease outbreaks like avian flu are highlighted by this study. While using social media exclusively for surveillance purposes may result in an inaccurate representation of the distribution of confirmed cases, it can be a useful tool for understanding public sentiment and concerns during an outbreak.

The study framework was performed on a micro-level, with regard to a singular disease type, timescale and location, which creates opportunities for additional analysis on a global level to build a greater training dataset. Successful adaptations of such methods have already been established, with work conducted from Robertson and Yee being a prime example of global level analysis [8].

It is critical to integrate social media as a surveillance tool with traditional surveillance methods and official data sources to maximise its efficiency. Furthermore, advanced data mining techniques and real-time analysis can improve the accuracy and timeliness of outbreak detection and response. Finally, a comprehensive and strong surveillance system is essential for efficiently monitoring and managing disease outbreaks in order to preserve public health.

## Ethics statement

Developer level access was permitted by Twitter and obtained in October 2019, granting administrative permission to access the raw twitter data. All data was anonymised prior to analysis. No user identifiable data was scraped, and all text was aggregated and analysed together, hence no individual can be identified from the results.

Ethical approval was granted by the University of Stirling's General University Ethics Panel (GUEP) and conformed to the research integrity policies.

All methods were carried out in accordance with relevant guidelines and regulations surrounding social media scraping and analysis provided by UK research and Innovation (UKRI). Further information can be found here: https://www.ukri.org/councils/esrc/guidance-for-applicants/research-ethics-guidance/internet-mediated-research/

## Consent for publication

Not applicable.

## Informed consent

The need for informed consent was waived by the University of Stirling's General University Ethics Panel (GUEP).

## Funding

Not applicable.

## Author contributions

All authors contributed to the study conception and design. Material preparation, data collection and analysis were performed by S.M. The first draft of the manuscript was written by S.M and all authors commented on previous versions of the manuscript. All authors read and approved the final manuscript.

## CRediT authorship contribution statement

**Samuel Munaf:** Conceptualization, Data curation, Formal analysis, Methodology, Validation, Visualization, Writing – original draft, Writing – review & editing. **Kevin Swingler:** Conceptualization, Formal analysis, Investigation, Methodology, Project administration, Resources, Software, Supervision, Validation, Visualization, Writing – original draft, Writing – review & editing. **Franz Brülisauer:** Conceptualization, Investigation, Methodology, Project administration, Supervision, Writing – review & editing. **Anthony O'Hare:** Conceptualization, Project administration, Supervision, Writing – review & editing. **George Gunn:** Investigation, Project administration, Supervision, Validation, Writing – review & editing. **Aaron Reeves:** Conceptualization, Project administration, Supervision, Validation, Writing – review & editing.

## Declaration of Competing Interest

The authors declare that they have no known competing financial interests or personal relationships that could have appeared to influence the work reported in this paper.

## Data Availability

All data was scraped from the public domain and can be requested by contacting the corresponding author.
